# Expectations for safety of nursing home residents and their family members during the COVID-19 pandemic: a qualitative study

**DOI:** 10.1186/s12912-023-01535-y

**Published:** 2023-10-06

**Authors:** Baojuan Cui, Hui Li, Yan Cheng, Jinmei Wang, Qiangsan Sun, Yuxiu Jia

**Affiliations:** 1 Department of Rehabilitation Medicine, The Second Hospital of Shandong University, Jinan, China; 2 Jinan Shande Nursing Home, Jinan, China; 3 Standardized training Department for residents, The Second Hospital of Shandong University, Jinan, 250033 China

**Keywords:** Expectations, Nursing home residents, Family members, Qualitative, COVID-19

## Abstract

**Background:**

COVID-19 has spread worldwide. Older adults are at the greatest risk of contracting and dying from the virus. Nursing homes are densely populated places for older adults who are generally vulnerable and at high-risk. Although Chinese nursing homes have been trying to protect their residents, the needs and expectations of the residents and their families have been ignored. This study aimed to promote the safety of NH residents, including their physical and psychological safety, and to meet their expectations during the COVID-19 pandemic in China.

**Methods:**

Data were collected through face-to-face semi-structured interviews with nursing home residents and focus group online interviews with family members between June 2021 and February 2022. Data analysis was performed using inductive content analysis.

**Results:**

16 residents and 24 family members were interviewed. Four themes with 10 sub-themes were identified from the participants’ descriptions. Their expectations were mainly focused on prevention and control measures for COVID-19, medical capacity of nursing homes, health literacy and expectations for some aged care policies.

**Conclusions:**

In the face of concerns about the impact of COVID-19 on nursing homes, we sought to bring firsthand perspectives to the forefront by interviewing residents and their family members about their expectations for safety to address this issue. Our findings provide important areas on which should be focused and may improve the sense of gain, happiness, and security of nursing home residents during the COVID-19 pandemic.

**Supplementary Information:**

The online version contains supplementary material available at 10.1186/s12912-023-01535-y.

## Background

The aging population has become a worldwide pressing issue, and China is no exception. In 2021, the proportion of the population over 60 years old in China reached 18.70%, an increase of 5.44% compared to 2010 [1].With the improvement of living standards and people’s awareness of health care, an increasing number of older adults have chosen to live in nursing homes (NHs) ,which marks the final phase of their lives [2]. And they expect less pain and more happiness, respect and dignity in this phase [3].

Since 2020, COVID-19 has spread worldwide. Older adults are at the greatest risk of contracting and dying from the virus. A case analysis reported a mortality rate of 8.0% in the 70–79 age group and 14.8% in the 80 years and older age group [4]. NHs are densely populated places for older adults who are generally vulnerable and at high-risk. Chinese NHs have implemented measures to reduce the risk of epidemic transmission, including social isolation, confinement, and strict quarantine [5]. These measures are easily accepted by the residents owing to their desire for safety. However, the fear caused by COVID-19 and some measures have negatively impacted NH residents [6]. Although NHs have been trying to protect their residents, the needs and expectations of the residents and their families have been ignored.

Previous research mainly showed the impact of COVID-19 on NH residents [7, 8], and little attention was paid to their wishes and needs during this special time. Residents are actually more concerned about and want to express their own expectations during unusual times [9]. Similarly, their families can also provide good suggestions for NH safety.

We explored the expectations regarding the safety of NH residents and their family members in the context of COVID-19 to better understand NH residents, meet their expectations and needs, and promote their physical and psychological health.

## Methods

### Aim

The aim was to promote the safety of NH residents, including their physical and psychological safety, and to meet their expectations and needs during the COVID-19 pandemic in China.

### Study design and sample

Semi-structured focus group interviews were conducted using a descriptive qualitative design. Participants, including NH residents and their family members, were recruited purposively from two NHs (range 260–300 beds) in Jinan, Shandong, China. Residents were recruited by nurses, and family participants were contacted by managers through video-based technology. The criteria for the residents were as follows: (a) being admitted to the NH for ≥ 3 months [3], (b) ≥ 60 years of age [1], (c) cognitively able to communicate normally with a score of more than 26 on the Mini-Mental State Examination [10], and (d) willing to share their thoughts voluntarily. Sampling of residents was considered adequate when data saturation was achieved. 16 residents and 24 family members participated in the study.

### Data collection

We collected data through face-to-face interviews with residents and online focus group interviews [11] with family members.

Semi-structured interview guides were developed based on prior literature [12, 13] and optimized through pilot interviews. The final guidelines for residents were as follows: (a) How is your life in the NH? (b) What changes have you experienced since the beginning of the COVID-19 pandemic? (c) What is your need during the COVID-19 pandemic? (d) What are your expectations for NH during the COVID-19 pandemic? The guidelines also included questions about the strengths and challenges of NH care. The residents were interviewed in their living rooms or NH gardens between November 2021 and February 2022. We began the interviews with their experiences at the NHs and encouraged them to speak freely. We told them that they could stop when they were tired and make another appointment. According to their physical condition and expression willingness, six residents were interviewed once, and ten were interviewed twice. Each interview lasted between 40 and 90 min and was recorded throughout. Nonverbal messages, including tone of voice, facial expressions, and body language, were also noted down.

Family members were divided into four groups of six each, and online focus group interviews were conducted from June to November 2021. C.B. and J.Y. conducted interviews.

The guidelines covered the following questions: (a) How well do you think you know residents’ wishes, needs and attitudes? (b) How do you assess the measures implemented by NH to reduce the risk of epidemic transmission? (c) What are your expectations and hopes for NH during the COVID-19 pandemic? (d) Do you have any suggestions for the NH? And what are they? Each interview lasted for approximately 90 min and was recorded using screen recording.

### Data analysis

After each interview, the recordings were transcribed into textual material within 24 h, which explored the emergent themes in subsequent interviews and identified when data saturation occurred until there was no new data.

Data analysis was conducted using inductive content analysis [14], including a process of three main phases: open-ended coding, category creation, and abstraction. In phase one, we read the written materials repeatedly to make overall sense and coded the text in an open way with notes and headings written in the text, as well as nonverbal information. We then read the text again, wrote down all the necessary headings to fully describe the content, and organized the headings to form a coding schedule. In phase two, the codes were categorized, and each category was named with feature words. Lastly, categories were abstracted according to the general description of the research topic; subcategories were classified as categories with similar themes, and categories were classified as the main categories. The data were independently coded by two researchers. Any questionable derivations were reconciled through research team meetings. Finally, all the researchers reviewed the data and findings. An example of this analytical process is presented in Table 1.

**Table 1:** Example of data analysis in the category“medical capacity”

Sub- Categories	Code	Meaning unit
Professional medical team	Traditional Chinese medicines	*Traditional Chinese medicine cupping has a very good effect on enhancing resistance. I think the resistance is very important in this special period. (R1)*
	Emergency and critical care ability	*My father suffers from multiple diseases and I hope the NH can let him live if he is infected with COVID-19. (FM5)*
Caregiver stability	Stop the turnover of caregivers	*The NH has changed three caregivers for me in two weeks, which makes me very uneasy. (R5)*
	Professional caregivers with knowledge of medicine and infectious disease	*I really hope that he (caregiver) is familiar with COVID-19, so that we have something to talk about at least. (R12)*
More humanistic care	Expect more attention to make up for the absence of their children	*I want everyone here to care about me (with shy smile). (R14)*
	To be visited more often by medical staff	*I hope doctors will check my physical condition so as to find out whether I am infected by COVID-19 in time. (R9)*
	More recreational activities	*I hope my mother can exercise regularly every day, and group exercise may make her happier. (FM23)*
An in-house psychological support service	Psychological counseling or intervention from professional psychologists	*Due to our absence and the epidemic, it is easy for her (his mother) to have psychological problems. Nursing home should do something for her. (FM14)*

### Ethical considerations

#### Ethical approval

was granted by the Research Ethics Committee of the Second Hospital of Shandong University. Electronic or on-paper written informed consent was obtained from all participants, and they all volunteered to participate in the study. They were free to express their opinions on how they expected to face the pandemic. They could refuse to answer any questions or withdraw from the study at any time. All recordings were kept under the principle of confidentiality.

### Rigor

A four-dimensional criterion was used to assess the rigor of the research [15]. Pilot interviews ensured the rationality of the interview outline. Interviews were conducted according to the residents’ conditions with familiar environment, which improved the credibility of the data. This study was conducted in the context of the global COVID-19 pandemic for transferability. Research team meetings and a review by all researchers increased the confirmation of findings.

## Results

### Participants

The characteristics of the 16 residents (represented as R1-R16) and 24 family members (represented as FM1-FM24) are shown in Tables 2 and 3. The median age of the residents was 76.3 years (range = 60.0–87.0 y), and all of them had chronic underlying medical disorders. They all had children, and only 30% had received higher education. The median age of the family members was 49.5 years (range = 29.0–67.0 y). Most were children, sons-in-law, or daughters-in-law of NH residents (87.4%), and 87.5% lived in the same city where the NH was located.

**Table 2:** Characteristics of residents (N = 16)

Characteristics	N (%)	Characteristics	N (%)
Gender		Marital status	
Female	9 (56.3)	Widowed	6 (37.5)
Male	7 (43.7)	Married	10 (62.5)
Age (years)		Education	
60–64	1 (6.1)	Higher education	5(31.3)
65–69	3 (18.8)	Secondary education	6(37.5)
70–74	3 (18.8)	Primary education	5(31.3)
75–79	3 (18.8)	Suffered from chronic underlying medical disorders	
80–84	4 (25.0)	Yes	10 (100.0)
≥ 85	2 (12.5)	Months since moving into a NH	
Number of children		3–12	3 (18.8)
1	11 (68.8)	13–24	6 (37.5)
2	3 (18.8)	25–36	4 (25.0)
3	1 (6.2)	≥ 37	3 (18.8)
4	1 (6.2)		

**Table 3:** Characteristics of family members (N = 24)

Characteristics	N (%)	Characteristics	N (%)
Gender		Relationship with the resident	
Male	11 (45.8)	Spouse	1 (4.2)
Female	13 (54.2)	Child or son-in-law or daughter-in-law	21 (87.5)
Age (years)		Grandchildren	1 (4.2)
20–29	1 (4.2)	Others	1 (4.2)
30–39	1 (4.2)	Place of residence and NH	
40–49	10 (41.6)	In the same city	21 (87.5)
50–59	7 (29.2)	In different cities	2 (8.3)
60–69	5 (20.8)	In different countries	1 (4.2)

Expectations of residents and their family members covered four themes and 10 sub-themes, which are shown in Table 4 and are described below.

**Table 4:** Summary of themes and subthemes

Themes	Subthemes
Prevention and control measures for COVID-19	Epidemic prevention consumables
	Cleaning and disinfection
	Access and visitation
Medical capacity	Emergency response ability
	Caregiver stability
	More humanistic care
	An in-house psychological support service
Building health literacy	Knowledge of infectious disease and health care for older adults
Expectations for some aged care policies	Community care center for older adults
	Raising public awareness of NH

## Themes

### Theme 1: prevention and control measures for COVID-19

#### Sub-theme 1: epidemic prevention consumables

Residents expressed concerns regarding the adequacy of protective materials in the NH. Each of them consumed at least two masks per day, which caused them to worry about the stock of masks. Some others considered whether there would be enough disinfectants and protective clothing for doctors and nurses if COVID-19 invaded. Families also described their concerns about the supply of protective materials such as masks and sanitizers. They all expected the NH to stock up enough materials to face the threat of COVID-19.

“Nurses ask us to change masks every four hours. The consumption of masks is so high that I hope the free supply will not be cut off.” (R3).

#### Sub-theme 2: cleaning and disinfection

Residents hoped that floors, beds, water dispensers, and other personal belongings could be disinfected as well as the public environments every day. A dedicated clothes disinfection device and harmless disinfectant with no irritating odor were also on their wishlist. Family members wanted the courier packages to be sterilized before entering the NH, and recommended placing quick-drying hand sanitizers in crowded places such as communication rooms, canteens, and exercise rooms.

“The virus is so terrible that I hope everything in the house is to be disinfected every day.” (R7).

“Express parcels are easily contaminated with viruses during mailing. The NH should disinfect the parcels before receiving them.” (FM2).

#### Sub-theme 3: access and visitation

Family members suggested that the NH adopt strict access restrictions. Couriers and material delivery personnel should be restricted to enter in, and items should be handed over without contact. They hoped that the NH workers, including medical staff and caregivers, would be housed in the NH during the pandemic. Even so, residents still expected their families to be allowed to visit them.

“I will miss my kids for sure as time goes by and I hope they can come often.” (R11).

“It is safest for people in the NH not to go out and people outside not to come in, including their staff.” (FM6).

### Theme 2: medical capacity

#### Sub-theme 1: emergency response ability

Residents were convinced that Chinese medicine would have a preventive effect on the COVID-19 infection, so they expected the NH to provide traditional Chinese medicines to enhance daily health care and increase their resistance. The ability of NH to provide emergency and critical care for COVID-19 patients was the biggest concern of family members. They said that their parents would struggle to resist infectious diseases with chronic underlying medical disorders. Therefore, they hoped that the NH would have a high-level medical aid team dealing with critical and severe cases.

“I believe that traditional Chinese medicine is more effective against COVID-19, even if it is used in healthcare.” (R4).

“……. I really hope the NH will save him when something urgent happens, at least let me see him for the last time. ” (FM11).

#### Sub-theme 2: caregiver stability

Residents reported a very high caregiver turnover. They needed to keep getting familiar with new caregivers and were prone to anxiety. Both residents and family members stated that some caregivers could only provide simple living care with little knowledge of basic medicine and infectious diseases, which was not conducive to limiting the spread of COVID-19.

“When I call my father’s caregiver to know dad’s condition, he doesn’t seem to understand any of the questions I ask about the illness.” (FM9).

“He (caregiver) wiped the ground with 84 disinfectant directly, instead of diluting it in a certain proportion.” (R13).

#### Sub-theme 3: more humanistic care

Residents said that the visitation restrictions for their family members made them yearn for more care and attention, so they expected doctors and nurses to visit them more often so that they could feel cared for and protected. They hoped that the NH would offer more recreational activities to distract them from missing their families.

“I want more smiles, more greetings and more love to help drive away my fear (of COVID-19).” (R10).

“I hope there will be more entertainments to make our life different, rather than immersed in missing my family because of the damn pandemic.” (R6).

#### Sub-theme 4: an in-house psychological support service

Residents indicated that the NH services were mainly limited to physical activities and disease treatments, with little psychological care. With fewer opportunities for family visits, some residents felt anxious, lonely or depressed while struggling with their relatives’ absence. Nearly half of the residents required psychological counseling or interventions. Family members hoped that professional psychologists would be employed by the NH to help the residents in need.

“My mother is prone to anxiety. The epidemic made her even more frightened and disturbed. I hope a psychologist can enlighten her.” (FM17).

### Theme 3: building health literacy

#### Sub-theme: knowledge of infectious disease and health care for older adults

Residents generally attached great importance to their own health. Being unfamiliar with internet, the knowledge of infectious diseases and healthcare of residents mainly came from TV programs or newspapers. They wanted to learn more about COVID-19, especially self-protection methods, the correct choice and wearing method of masks, the transmission route of the new coronavirus, and the clinical symptoms of COVID-19. They expected that the NH would hold regular health lectures to broaden their medical knowledge and enrich daily activities at the same time. Family members hoped the NH to popularize the knowledge of rehabilitation and exercise of older people so that they could better understand the conditions of their parents.

“I would like to know more clearly what the symptoms will be if I get infected with COVID-19 so that I can receive treatment in the early stages.” (R16).

“If only doctors or nurses could give regular lectures on infectious diseases, so that I can better protect myself.” (R9).

“I hope the NH could popularize medical and health knowledge to us, so that I will talk with my mother about physical recovery more than just about eating and sleeping.” (FM24).

### Theme 4: expectations for some aged care policies

#### Sub-theme 1: community care center for older adults

Residents described that most NHs were built in the suburbs. The long distance between NH and their homes made them feel psychologically alienated. They expressed that if a NH could be established in their communities, the familiar environment would encourage them to give full play to their initiatives and boost their psychological comfort even in the face of COVID-19.

“It took us forever to drive here for the first time. The further I was away from home, the more scared I felt.” (R2).

“I really wish the NH is close to home. I would feel much cared for even when family visits were forbidden during the pandemic.” (R8).

#### Sub-theme 2: raising public awareness of NH

All the participants appreciated the integrated model of combining medical services and aged care. However, influenced by Chinese traditional culture, both of them also expected the government to publicize the advantages of NH widely, so that people could have a correct understanding of NH, and which might avoid making them have to deal with not only the fear of the pandemic, but also the criticism of their relatives.

“Since I sent my mother to the NH, my relatives living in the countryside have accused me of failing to fulfill my duty of supporting my mother.” (FM16).

“People say that I have been abandoned by my daughter (wry smile), but my life in the NH is full and happy.” (R15).

## Discussion

COVID-19 is a highly contagious disease leading to an ongoing epidemic that brings challenges to NHs [16]. Although NHs have taken measures to protect their residents, the expectations for the safety of residents including family members provide a viewpoint for addressing and understanding this issue, which focused on prevention and control measures for COVID-19, medical capacity of NH, building health literacy and expectations for some aged care policies. If their expectations can be met, their fear caused by COVID-19 may decrease.

### Prevention and control work is the focus of expectations of NH residents and their families

Residents and their families were mainly concerned about the effectiveness of prevention and control measures. The subthemes of these measures fully show that residents want NHs to keep them safe during the pandemic. Epidemic prevention consumables such as masks and disinfectants can not only help residents isolate the virus, but also reduce their worry and ease their anxiety. NHs should reserve emergency protective materials, obtain multiple channels for resources, and formulate distribution strategies when these materials are insufficient.

Cleaning and disinfecting of the environment could effectively control the spread of COVID-19 [17]. However, this study found that the NH staff lacked disinfection knowledge and sufficient executive power, which was not conducive to controlling the spread of COVID-19. The result might be attributed to less training in NHs; therefore, NHs should be responsible for training staff in disinfection. While doing a good job of disinfection, NHs should also grasp the sterilization intensity. Because excessive disinfection not only wastes the operating costs, but also exposes the residents to the environment of “potentially excessive sterilization” [18].

Personnel mobility is a difficult problem in epidemic prevention and control. Staff access and family visitations are extremely risky factors for NH because these could also be the entrances to the virus. If the staff could be accommodated in NHs, the risk of staff moving between facilities and their homes might be reduced. Visitation restrictions could interrupt the spread of COVID-19 to some extent, but also heighten mental health challenges among NH residents, as confirmed by Raciti [19]. NHs may establish flexible and diversified visitation systems, such as video chatting, to ease the older adults’ yearning for their families.

### Medical capacity is another focus for the residents and their families

NH residents have high healthcare needs [20]. Owing to their weak immunity and resistance, they have become susceptible and vulnerable to COVID-19. In the face of the epidemic, their vulnerability becomes obvious, including physical, psychological and emotional aspects [21]. They want to stay healthy and are afraid to face loneliness and disease more than ever before. Therefore, they have higher expectations for the emergency response and humanistic care.Traditional Chinese medicine healthcare services, such as massage, moxibustion, cupping, and acupuncture, are very popular in Chinese NHs [22], and residents in this study also expressed this expectation.

Turnover or shortage of caregiver staff is a pervasive challenge for many NHs [23]. Their lack of medical knowledge is also a common problem in China [24], which may be related to wages, social status, and staff feeling appreciated and supported [25]. A professional caregiver team could improve the life quality of residents, and solve the contradiction between supply and demand between the residents and the NH. Therefore, NHs should be staffed with adequate caring staff, which also helps to prevent and control infection, eliminate the residents’ anxiety, and bring a sense of security to them. Adequate training and strategies to acclimate new caregiver staff to residents’ preferences can also ensure safe and high-quality resident care [25].

Approximately 58.3% of NH residents have depressive symptoms [26] exacerbated by the epidemic. This may have been due to psychological panic and restrictions on family visits. Therefore, NHs need to establish professional medical teams, such as multidisciplinary health care teams, which can not only meet the residents’ needs to provide daily medical care and an in-house psychological support service, but also have the ability to treat COVID-19 as well as its complications [27].

### Improve the health literacy of residents and their family members

Both residents and their families expected to learn about knowledge of infectious diseases and healthcare to improve their own health literacy. Health literacy refers to the ability of people to obtain health-related information, make health-related evaluations, and improve disease prevention and health decisions [28]. Because of limited access, the information obtained by residents through television or by the people around them is relatively piecemeal and difficult to systematize. Due to diseases or physical aging, the functions of hearing, eyesight, memory, and attention of the residents are decreased, thereby affecting the effect of health education. Additionally, with the development of COVID-19 epidemic, protective knowledge is constantly being updated. Therefore, older adults belong to the weak health literacy group. In terms of knowledge of infectious diseases, targeted education should be conducted on residents’ knowledge of blind spots and their shortcomings. NHs could carry out popular science publicity through lectures, posters, broadcasts, and sitcoms to improve their health literacy.

### Aged care services need policy support

With advantages in medical care, NHs should be attractive to older adults. However, it seems to be ignored by a large number of Chinese people. Studies have found that awareness of the model of “combining medical service and aged care” among middle-aged and older adults is low [29]. Moreover, due to traditional Chinese culture, many people are prejudiced against institutionalized care. Therefore, the Chinese government should publicize the characteristics and advantages of NHs to allow more people to accept them.

Community NH is an institution established in the community providing residents with health care, medical treatment, and aged care services [30]. Due to familiar local conditions and customs, community-based interventions can effectively reduce the psychological vulnerability [31]. We suggest building community NHs to meet the multiple needs of medicine, aged care and emotional family support for older people.

In the context of pandemic, the NH residents and their families have many expectations. Some can be realized through the efforts of the NHs, such as storing sufficient consumables, improving medical capacity, and building health literacy for residents and their families. However, some require support from government departments and the whole society, such as the establishment of community care centers. Moreover, improving the emergency response ability and caregiver stability of NH would take some time. Our findings explore the expectations of NH residents and their families and suggest the importance of the psychological and emotional needs of older people in the context of COVID-19. Moreover, this result reinforces the ongoing need to regulate NH development to ensure that aged care services are in accordance with the expectations of their residents and family members.

## Limitations

Interviews were conducted with nursing home residents and their family members from a single geographical region, so the results might be geographically limited. Were the study conducted in multiple regions, the findings would be more representative. The absence of police-makers is also a limitation. If they can participate in the study, more comprehensive results will be generated. The potential for response bias is another limitation. This bias may be attributed to the potential recruitment method for the interview partners. Because interviewees from nursing homes were more likely to be close to residents who had already discussed the subject or who were willing and able to provide information.

## Conclusions

In the face of concerns regarding the impact of COVID-19 on nursing homes, we sought to bring firsthand perspectives to the forefront by interviewing residents and their family members about their expectations to address this issue. Our findings provide important areas on which should be focused and may improve the sense of gain, happiness, and security of NH residents during the COVID-19 pandemic.

### Electronic supplementary material

Below is the link to the electronic supplementary material.

Supplementary Material 1

## Data Availability

Most data generated or analyzed during this study are included in this published article, and other data could be obtained from the corresponding author.

## Abbreviations

NH: Nursing Home
