# Supplementary material for: Expectations for safety of nursing home residents and their family members during the COVID-19 pandemic: a qualitative study
Source: BMC Nurs. 2023 Oct 6;22:365. doi: 10.1186/s12912-023-01535-y (PMC10559523; doi:10.1186/s12912-023-01535-y)
Supplement: Supplementary file 1 — Supplementary Material 1 [file 12912_2023_1535_MOESM1_ESM.docx]

**The interview guides:**

1. The guidelines for the resident interviews contained the following key questions:

(a) How is your life in the NH?

(b) What changes have you experienced since the beginning of the COVID-19 pandemic?

(c) What is your need during the COVID-19 pandemic?

(d) What are your expectations for NH during the COVID-19 pandemic?

(e) What do you think are the main challenges facing the NH at present?

(f) What other improvements do you think the NH needs to make?

2. The guidelines for online focus group interviews with family members covered the following questions:

(a) How well do you think you know residents’ wishes, needs and attitudes?

(b) How do you assess the measures implemented by NH to reduce the risk of epidemic transmission?

(c) What are your expectations and hopes for NH during the COVID-19 pandemic?

(d) Do you have any suggestions for the NH? And what are they?
